# Investigation of Trace and Macro Element Contents in Commercial Cat Foods

**DOI:** 10.1002/vms3.70123

**Published:** 2024-12-11

**Authors:** Bengü Bilgiç, Duygu Tarhan, Fatma Ateş, Çağla Parkan Yaramiş, Lora Koenhemsi, Mehmet Erman Or

**Affiliations:** ^1^ Department of Internal Medicine Faculty of Veterinary Medicine Istanbul University‐Cerrahpasa Istanbul Turkey; ^2^ Department of Biophysics School of Medicine Bahcesehir University Istanbul Turkey; ^3^ Department of Biophysics School of Medicine Bezmialem Vakif University Istanbul Turkey; ^4^ Equine and Training Program, Vocational School of Veterinary Medicine Istanbul University‐Cerrahpasa Istanbul Turkey

**Keywords:** cat, elements, food, minerals, prescription

## Abstract

**Background:**

Nutritional profiles and guidelines are determined by various associations to ensure optimum health of cats and provide pet food manufacturers nutritional recommendations to ensure the well‐balanced and nutritionally adequate pet food.

**Objectives:**

It was aimed to determine some trace elements and macro minerals in prescription and non‐prescription dry cat foods and compare the contents with the suggested guidelines to evaluate the potential in‐compliance.

**Methods:**

A total of 96 dry cat foods were evaluated. Cu, Fe, Mn, Se, Zn, Mg, Ca and P concentrations were analysed in prescription dry cat foods developed for gastrointestinal diseases—GI (*n* = 18), renal diseases—R (*n* = 15), urinary diseases—U (*n* = 8), obesity—O (*n* = 10) and non‐prescription—N‐P (*n* = 45) foods from different flavours and brands by ICP‐OES.

**Results:**

Ten precent of the O group exceeded the upper limit, and 15.5% of the N‐P group failed to provide a nutritional minimum level. 4.44% of prescription foods were below the minimum recommended Mn level according to the European Pet Food Industry Federation (FEDIAF), and 66% of N‐P foods were below the minimum adult maintenance value recommended by the Association of American Feed Control Officials (AAFCO). One hundred percent non‐compliance with the guidelines in the GI and U groups was recorded. 22.2% of the GI group was lower, and 5.55% of the GI group was higher than the recommended levels by FEDIAF, which reflects the 27.7% in compliance. While all the prescription foods have 100% compliance with the determined minimum level of guidelines, 11.1% of N‐P foods were below the minimum recommended level. In compliance with the guidelines for Ca was noted in the O and U groups (10% and 12.5%, respectively). The P ratio in all prescription food groups except for GI was below the minimum level determined by AAFCO.

**Conclusions:**

In all food groups, mean Cu, Fe, Mn, Mg, Ca and P concentrations were between the minimum and maximum limits recommended by FEDIAF. However, the mean Se level was above the upper legal limit in all food groups, and the Zn level was below the minimum recommended level in the N‐P food group. Besides the mean values, a high number of foods in each group show a broad in‐compliance with the guidelines.

## Graphical abstract

Some element and mineral values showed significant differences between prescription and non‐prescription dry cat foods. Since it was observed that the high number of foods exceeded the upper limit or below the minimum recommended level by FEDIAF, AAFCO or NRC, both regulatory guidelines and the elemental composition of commercial cat foods need reconsideration.

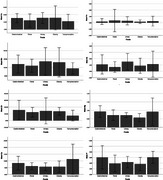

## 1 Introduction

Diet is the main source of trace elements and minerals. Adequate dietary intakes of these components are essential to maintain normal physiological status and health in cats (Underwood 1977). They are absorbed by intestinal epithelial cells and transported to tissues as cofactors or structural protein components, which play an important role in several physiological functions (Summers, Stockman, and Larsen 2022). Since the liver, kidney and other tissues are the primary reservoirs for trace elements, excessive intake may lead to an accumulation in many tissues. On the other hand, considering its essential role in many metabolic processes, inadequate dietary intake may also lead to various health problems (Goldhaber 2003). Nutritional guidelines have been provided by the food authorities to produce nutritionally balanced pet foods (FEDIAF 2021; National Research Council et al. 2006; AAFCO 2015).

The element and mineral content of complete pet foods, nutritional profiles and guidelines are determined by various associations to ensure the optimum health of cats and dogs. The European Pet Food Industry Federation (FEDIAF) compiled nutritional guidelines for complete and complementary pet food for cats and dogs, which provides pet food manufacturers with nutritional recommendations to ensure the production of well‐balanced and nutritionally adequate pet food (FEDIAF 2021). In addition, the National Research Council (NRC) Committee on Animal Nutrition published general considerations regarding feed ingredients, diet formulation and feed processing (National Research Council et al. 2006). Also, according to the Association of American Feed Control Officials (AAFCO), ‘Dog and Cat Food Nutrient Profiles’ were designed to establish practical minimum and some maximum nutrient concentrations for dog and cat foods formulated from commonly used, non‐purified, complex ingredients (AAFCO 2015).

Trace elements play an active role in maintaining metabolic functions and enzymatic reactions, regulating the immune system and the redox balance in companion animals. Of these, copper (Cu) acts as a cofactor in several enzymatic reactions that are necessary for the growth and development of the cells (Gaetke and Chow 2003). Iron (Fe) has several essential functions, such as haemoglobin, myoglobin, neurotransmitter and myelin production, immune regulation, energy metabolism, DNA and RNA synthesis and several enzymatic reactions (McCown and Specht 2011). Manganese (Mn) is an important micronutrient for cellular, enzymatic and neurologic mechanisms in both animals and humans (Horning et al. 2015). Adequate intake of selenium (Se) is necessary for growth, fertility, antioxidant defence and thyroid homeostasis in cats (Simcock, Rutherfurd, and Hendriks 2002). Zinc (Zn) metalloenzymes and metalloproteins are involved in several cellular metabolisms, redox signalling, gene expression, membrane structure and formation (Cummings and Kovacic 2009). Calcium (Ca) and magnesium (Mg) are the essential cations that play a crucial role in ion homeostasis, mitochondrial membranes, oxidative phosphorylation and ATP production (Humphrey, Kirby, and Rudloff 2015). Similarly, phosphorus (P) is one of the essential macro minerals that play a role in many metabolic processes such as energy metabolism, bone and tooth formation, acid‐base balance, electrolyte transportation and several enzymatic reactions (Laflamme et al. 2020). In this study, it was aimed to determine some trace elements and macro minerals in prescription and non‐prescription dry cat foods and compare the element contents with the suggested guidelines to evaluate the potential in‐compliance.

## 2 Materials and Methods

This study was carried out at Istanbul University Cerrahpaşa, Faculty of Veterinary Medicine, Department of Internal Medicine and Cerrahpaşa Faculty of Medicine, Department of Biophysics. Non‐prescription dry cat foods from different brands and flavours of various companies sold on the market and prescription dry cat foods from different brands and flavours of various companies sold in only veterinary clinics were collected. In the study, a total of 96 dry cat foods were evaluated. Prescription diet groups included gastrointestinal dry cat foods (GI) developed for gastrointestinal diseases (*n* = 18), renal dry cat foods (R) developed for renal diseases (*n* = 15), urinary dry cat foods (U) developed for urinary system diseases (*n* = 8) and obesity dry cat foods (O) developed for the obesity management (*n* = 10). All adult prescription foods are introduced as premium, high‐quality, complete and balanced, formulated and manufactured in Italy and Spain. In all prescription diets, analytical constituents range as follows: protein 24%–42%, fat 8.7%–20.5%, crude fibre 2%–11.7% and coarse ash 7%–8.8%, with the composition of dehydrated wheat, fish, pork, turkey and poultry and hydrolysed fish and animal proteins.

The non‐prescription food group (N‐P) consists of different flavours and tastes developed for healthy cats (*n* = 45). In this group, all foods are labelled as premium, high‐quality, complete and balanced, formulated and manufactured in Italy and Turkey. In all non‐prescription diets, analytical constituents range as follows: protein 26%–44%, fat 9%–26%, crude fibre 1.8%–5.5% and coarse ash 8.3%–12%, with the composition of dehydrated fish, turkey, poultry and vegetable, hydrolysed fish and animal proteins.

### 2.1 Macro–Micro Element Analysis

Cu, Fe, Mn, Se, Zn, Mg, Ca and P concentrations were analysed from dry food samples with an inductively coupled plasma‐optical emission spectrometer (ICP‐OES), Thermo iCAP 6000 series. Suitable wavelengths for each element were selected for analysis (Table 1).

**TABLE 1:** Wavelengths (nm) of each element for ICP‐OES analysis.

Cu	Fe	Mn	Se	Zn	Mg	Ca	P
324.754	259.940	257.610	196.090	206.200	285.213	317.933	177.495

To analyse the element concentrations from the collected food samples, which came from multiple batches of each type of food, 4 samples were prepared, and the average values were calculated. Two milliliters of nitric acid (HNO_3_; 65% concentrated; Merck) was added to the food samples and melted in an oven at 200°C. After 1 mL of perchloric acid (HClO_4_; 60% concentrated; Panreac) was added to the nitric acid food mixture, the sample cooled at room temperature and vortexed. Distilled water was added to the samples, and the total volume was completed to 13 mL (Altunatmaz et al. 2017; Tarhan and Dursun 2022). It was vortexed again and analysed in the ICP‐OES device. The three‐point calibration was carried out using the standard solutions and blank solutions as reference points. Consistent and linear calibration curves were produced for analysis, and the correlation coefficient of the calibration curve was determined for each measured element. The recovery of the analysed quality control was between 96% and 102%. Standard solutions of all elements were prepared utilising solutions in as a blank solution and then analysed. Elemental concentrations in food samples prepared for analyses were determined using these standard curves. The ICP‐OES device for the determination of elements utilised parameters a plasma gas flow rate of 15 L/min, an argon flow rate of 0.5 L/min, a sample flow rate of 1.51 L/min, a peristaltic pump speed of 100 rpm and an RF power set at 1150 W. All samples were analysed on the same day and with the same calibration to minimise the factors affected by temperature, humidity and device calibration (Cihan et al. 2023; Or et al. 2024). Each measurement was performed in triplicate, and the results were averaged for analysis. Trace element concentrations were reported as mg/g or µg/g of wet weight of the samples.

### 2.2 Statistical Analysis

Data were analysed using the SPSS Statistics 25 program. Data sets were assessed for normality using the Shapiro–Wilk test. Analysed Cu concentrations were compared among the other food groups using a one‐way analysis of variance with Tukey's post hoc analysis. Analysed Fe, Mn, Se, Zn, Mg, Ca and P concentrations were compared using Kruskal–Wallis with Dunn's multiple comparisons test. With the Bonferroni correction, a value of *p* < 0.05 was considered significant. Results were expressed as mean ± standard deviation.

## 3 Results

The concentrations of some trace elements and macro minerals in prescription and non‐prescription dry foods for cats are reported in Table 2.

**TABLE 2:** Comparison of some element concentrations for prescription and non‐prescription food groups with minimum and maximum recommended levels by FEDIAF in 100 g dry matter (FEDIAF 2021).

			Mean measured element concentrations (per 100 g DM)				
Elements	Minimum recommended level for adults (per 100 g DM)	Maximum recommended level for adults (per 100 g DM)	GI (*n* = 18)	R (*n* = 15)	U (*n* = 8)	O (*n* = 10)	N‐P (*n* = 45)
Cu (mg)	0.50	2.80 (L)	1.509^a^	1.184^ab^	1.531^ab^	1.544^a^	1.065^b^
Fe (mg)	8.00	68.18 (L)	35.9	68.6	59.4	31.2	49.6
Mn (mg)	0.50	17.00 (L)	2.799^a^	2.407^ab^	3.427^a^	3.218^a^	1.774^b^
Se (µg)	21.00	56.80 (L)	216.9^ab^	135.3^a^	304.4^b^	176.1^ab^	227.8^ab^
Zn (mg)	7.50	22.70 (L)	15.58^a^	12.33^a^	14.24^ab^	13.63^a^	7.259^b^
Mg (g)	0.04	N/A	0.093^a^	0.071^ab^	0.071^ab^	0.057^b^	0.092^a^
Ca (g)	0.40	N/A	0.839^ab^	0.615^a^	0.569^a^	0.572^a^	1.130^b^
P (g)	0.26	N/A	0.603^bc^	0.386^a^	0.436^abc^	0.370^a^	0.606^b^

*Note*: ^a,b,c^Different letters in the same line indicate statistically significant differences.

Abbreviations: DM, dry matter; GI, gastrointestinal; L, legal limit; N/A, not available; N‐P, non‐prescription; O, obesity; R, renal; U, urinary.

**FIGURE 1:**
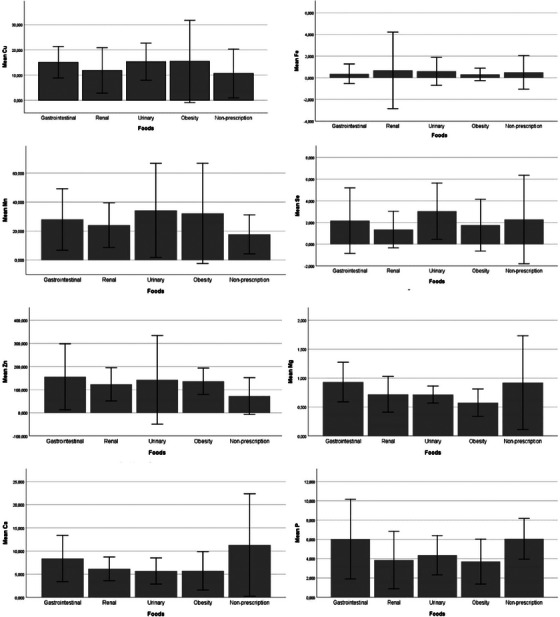
Bar graphs of the mean concentration of each element and mineral in food groups.

In comparison to prescription and non‐prescription foods, the Cu value in the GI and O groups was statistically higher than in non‐prescription foods (*p < *0.05); however, no significant difference was observed in R and U groups compared to other prescription and non‐prescription foods (*p *> 0.05). No statistically significant difference in Fe concentration was observed between all prescription and non‐prescription food groups (*p *> 0.05). It was determined that the mean Mn concentration in the GI (*p < *0.01), U (*p < *0.05) and O (*p < *0.05) groups was statistically higher compared to the N‐P group. The concentration of Se was statistically lower in the R group compared to the U group (*p < *0.05). The mean Zn level was statistically higher in the GI (*p < *0.001), R (*p < *0.05) and O (*p < *0.01) groups compared to the N‐P group (Table 2; Figure 1).

The macro minerals such as Mg, Ca and P were measured in prescription and non‐prescription dry foods. In the O group, mean Mg concentration was measured statistically lower than the N‐P and GI groups (*p < *0.01). It was determined that the mean Ca level in the N‐P group was significantly higher compared to R, U and O groups (*p < *0.001). The mean P value in the O and R groups was significantly lower than the GI and N‐P groups (*p < *0.01) (Table 2; Figure 1).

## 4 Discussion

### 4.1 Copper

Adequate dietary Cu intake is essential to maintain normal physiological status and health in cats. In our study, the highest and lowest amount of Cu was measured in the O and N‐P food groups. Also, in N‐P foods, Cu concentration was statistically lower than in GI and O food groups (*p *< 0.05). Since Cu is an active structure of copper/zinc superoxide dismutase (Cu/Zn SOD), which protects cells from damage caused by superoxide ions, moderate amounts of Cu are important for avoiding oxidative damage. Nevertheless, excess Cu can lead to imbalances in the oxidant/antioxidant system through catalysing reactive oxygen species (ROS) and reactive nitrogen species (Osredkar and Sustar 2011). In human medicine, many studies reported a significantly higher serum Cu level in obese individuals than in healthy controls (Yang et al. 2019; Gu et al. 2020; Wu et al. 2023; Ge, Liu, and Liu 2020). However, in veterinary medicine, one study reported insignificant serum Cu levels between obese and healthy dogs (Cihan et al. 2023). Considering several supportive reports regarding high Cu levels and obesity in human medicine, cat foods for obesity management may be expected to lower Cu formulations. Malabsorption of Cu associated with enteropathies in many gastrointestinal system disorders may contribute to the Cu deficiency. Therefore, in gastrointestinal prescription pet foods formulated for gastrointestinal health issues, adequate Cu levels are expected (Kanikowska et al. 2015). Cu deficiency–related impaired haematopoiesis, incomplete nervous system development, immune dysfunction, keratinisation and pigmentation defects have been suggested (Altarelli et al. 2019). Excessive hepatocellular Cu‐associated hepatopathy is well known. Hepatic copper accumulation resulting in copper‐associated chronic hepatitis and cirrhosis was reported in cats (Haynes and Wade 1995; Meertens, Bokhove, and Van den Ingh 2005).

In the study, measured mean Cu concentrations in both prescription and non‐prescription food groups were between the recommended minimum and maximum levels by FEDIAF (Table 2). In addition, a minimum 5 mg/kg adult maintenance Cu level per kg DM was determined by AAFCO (2015). All the food groups in the study were above the specified lower limit (Table 3). The NRC has recommended an optimum Cu concentration of 5 mg per kg dry matter for feline formulas (National Research Council et al. 2006) (Table 4). The safe upper limit was not specified in the guideline. In the GI, R and U groups, all foods complied with the guidelines (41/41). However, in the O group, 1 of 10 foods exceeded the upper limit (10%) and in the N‐P group, 7 of 45 (15.5%) foods failed to provide nutritional minimum level.

**TABLE 3:** Comparison of mean element concentrations for prescription and non‐prescription food groups with minimum recommended levels determined by AAFCO (AAFCO 2015).

Elements	Adult maintenance minimum	Mean measured element concentrations (per kg DM)				
		GI (*n* = 18)	R (*n* = 15)	U (*n* = 8)	O (*n* = 10)	N‐P (*n* = 45)
Cu (mg/kg)	5	15.09	11.84	15.31	15.44	10.65
Fe (mg/kg)	80	359	686	594	312	496
Mn (mg/kg)	7.6	27.99	24.07	34.27	32.18	17.74
Se (mg/kg)	0.3	2.169	1.353	3.044	1.761	2.278
Zn (mg/kg)	75	155.8	123.3	142.4	136.3	72.59
Mg (%)	0.04	0.093	0.071	0.071	0.057	0.092
Ca (%)	0.6	0.839	0.615	0.569	0.572	1.130
P (%)	0.5	0.603	0.386	0.436	0.370	0.606

Abbreviations: DM, dry matter; GI, gastrointestinal; N‐P, non‐prescription; O, obesity; R, renal; U, urinary.

**TABLE 4:** Comparison of mean element concentrations for prescription and non‐prescription food groups with minimum requirement, adequate intake and safer upper limit levels determined by National Research Council (National Research Council et al. 2006).

					Mean measured element concentrations (per kg DM)				
Elements	Minimum requirement (per kg DM)	Adequate intake (per kg DM)	Recommended allowance (per kg DM)	Safer upper limit (per kg DM)	GI (*n* = 18)	R (*n* = 15)	U (*n* = 8)	O (*n* = 10)	N‐P (*n* = 45)
Cu (mg)	N/A	5.0	5.0	N/A	15.09	11.84	15.31	15.44	10.65
Fe (mg)	N/A	80	80	N/A	359	686	594	312	496
Mn (mg)	N/A	4.8	4.8	N/A	27.99	24.07	34.27	32.18	17.74
Se (µg)	N/A	300	300	N/A	2169	1353	3044	1761	2278
Zn (mg)	N/A	74	74	> 600	155.8	123.3	142.4	136.3	72.59
Mg (mg)	200	N/A	400	N/A	932	719	716	574	921
Ca (g)	1.6	N/A	2.9	N/A	8.397	6.158	5.694	5.724	11.304
P (g)	1.4	N/A	2.6	N/A	6.032	3.862	4.361	3.701	6.067

Abbreviations: DM, dry matter; GI, gastrointestinal; L, legal limit; N/A, not available; N‐P, non‐prescription; O, obesity; R, renal; U, urinary.

Davies et al. reported that a number of foods, particularly wet food, were either above or below FEDIAF guidelines for Cu (Davies et al. 2017). In another study investigating the element status in dog foods and its compliance with guidelines, similar to our results, a statistically significant increase in Cu level in gastrointestinal dry dog food compared to N‐P food was observed (Or et al. 2024). In another recent study, the Cu composition of commercial dry dog foods was measured as 15.0 ± 7.4 mg/kg dry matter. In a similar study on dry cat foods, the mean Cu concentration was measured as 6.0 mg/1000 kcal (Summers, Stockman, and Larsen 2022). The mean Cu concentration of some pet foods commercially available in Turkey was determined in the range of 3.33–16.6 µg/g.

### 4.2 Iron

Iron is an essential component of several biochemical pathways such as haemoglobin and myoglobin formation, neurotransmitter and myelin production, collagen formation, immune system function, energy metabolism, DNA and RNA synthesis and many enzyme systems. While Fe deficiency results in anaemia, Fe overload may cause organ dysfunction secondary to iron‐induced injury (McCown and Specht 2011). The dietary Fe requirement for adult cats is estimated at 80 mg/kg dry matter (Dzanis 1994). Regarding Fe bioavailability, a study reported only 20% availability for cats (Henry and Miller 1995). In another study, haemoglobin Fe bioavailability was 70% for cats (Fly and Czarnecki‐Maulden 2000). In our study, although the lowest and highest Fe were measured in O and GI food groups, respectively, no statistically significant difference was observed between the prescription and non‐prescription foods.

According to the FEDIAF guideline, minimum and maximum Fe limits were determined as 8–68.18 per 100 g DM. Except for the R group, all prescription and non‐prescription foods were between the recommended ranges in this study. However, in the R group (68.6 per 100 g DM), the Fe level exceeded the upper legal limit slightly (Table 2). Although the safer upper limits were not stated in the NRC and AAFCO guidelines, compliance was noted for the minimum or adequate intake amounts of all groups (Tables 3 and 4). Iron deficiency anaemia is very common in renal diseases such as chronic kidney disease (Gafter‐Gvili, Schechter, and Rozen‐Zvi 2019). Feline renal diets were commonly recommended by veterinarians in cases of chronic kidney disease in cats. Considering the relationship between chronic kidney injury and iron deficiency due to an inability to utilise body iron stores for erythropoiesis because of the decreased erythropoietin production by damaged kidneys, a high Fe formula might be expected in the renal diets, which is consistent with our results. Consequently, all foods in the study complied with guidelines for Fe due largely to the exceptionally wide acceptable range.

In a study that evaluated the Fe concentrations in commercial dry foods formulated for healthy cats, the measured median Fe level was 61.4 mg (29.8–104.5), which is below the minimum limit recommended by AAFCO (Summers, Stockman, and Larsen 2022). In another study, measured Fe ranges were determined as 23.9–71.1 µg/g dry weight in pet food samples. In the GI group, 1 of 18 foods (5.55%) was below the minimum recommended level by FEDIAF, and in the N‐P group, 8 of 45 foods exceeded the upper legal limit (17.77%) (Duran, Tuzen, and Soylak 2008).

### 4.3 Manganese

Manganese is considered an essential trace element because of its important functions in enzyme systems. Its deficiency causes severe health problems such as skeletal abnormalities, reproductive disorders and alterations in glucose and lipid metabolism; its toxicity results in neurotoxicity and neurodegeneration in domestic animals (Finley and Davis 1999; Wolf, Hoffman, and Southern 2023; Pfalzer and Bowman 2017). Although the lowest Mn concentration was measured in the N‐P food group since Mn has a wide safety range, all of the food groups in the study included between the recommended minimum and maximum ranges of FEDIAF (Table 2). Of 45 N‐P foods, 2 (4.44%) were below the minimum recommended Mn level by FEDIAF, and 3 of 45 N‐P foods (6.66%) were below the minimum adult maintenance value recommended by AAFCO. All of the prescription food was compliant with the guidelines.

In a study, reference ranges of Mn concentrations of some pet foods were reported as 3.28–24.4 µg/g (Duran, Tuzen, and Soylak 2008). Davies et al. reported the mean concentration of Mn in dry cat foods as 59.6 µg/kg DM, which complies with all guidelines (Davies et al. 2017). Also, the mean Mn values in selected prescription and non‐prescription dog foods were compared, and similar to our results, the lowest mean concentration was noted in non‐prescription dog foods (Or et al. 2024).

### 4.4 Selenium

Selenium is an essential trace element in small animals since it is involved in many physiologic processes such as antioxidant protection, thyroid hormone metabolism and immune function (Kiełczykowska et al. 2018; Hooper, Backus, and Amelon 2018; O'Brien 2010). Intestinal Se absorption is dependent on the chemical form of the element. Inorganic forms of Se include hydrogen selenide (H_2_Se), elemental selenium (Se^0^), selenite (${\mathrm{Se}}_{3}^{-2}$) and selenate (${\mathrm{Se}}_{4}^{-2}$); organic forms are selenomethionine and selenocysteines (Jacques 2001). However, the European Scientific Committee for Food concluded that all dietary forms of Se can be absorbed efficiently. The reports of the US Food and Nutrition Board suggested that selenomethionine has the highest bioavailability (> 90% absorbable), approximately 100% of selenite and > 50% of selenite is absorbed (Fairweather‐Tait, Collings, and Hurst 2010). On the other hand, Se concentration in the diet and other dietary factors such as heavy metal contents and heat can affect the bioavailability (Todd, Thomas, and Hendriks 2012). As a Se source, most of the pet foods included sodium selenite. However, Se deficiency‐related disorders, such as Keshan disease in humans; and muscular and skeletal disorders in dogs and farm animals (Zentrichová, Pechová, and Kovaříková 2021; Chen 2012), Se deficiency–related disorders and Se toxicity have not been reported in cats. In addition, it was suggested that the cats have higher plasma Se concentrations compared to other species (Foster et al. 2001).

Compared to other animals, it was reported that plasma Se concentrations are quite high, which suggests that cats may tolerate relatively high dietary Se concentrations (Foster et al. 2001; Forrer, Gautschi, and Lutz 1991). In our study, the lowest concentration of Se was measured in the R group, and statistically lower than the U group (*p* < 0.05). In chronic kidney disease, glutathione peroxidases (GSH‐Px) play an important role in ROS. Se is required as a cofactor for GSH‐Px synthesis in the kidney (Zachara 2015). Therefore, an adequate amount of Se contributes to the antioxidant defence during kidney injury. However, in our study, the Se content of all food groups was higher than the maximum level recommended by FEDIAF and higher than the recommended allowance by NRC (Tables 2 and 4). Although a wide range of Se results were obtained in this study, 100% non‐compliance with the guidelines in the GI and U groups was recorded. Thirteen of 15 foods in the R group (86.6%), 8 of 10 foods in the O group (80%) and 38 of 45 foods in the N‐P group (84.4%) were higher than the determined upper limit. Similarly, Davies et al. reported that 76% of wet foods exceeded the legal maximum for Se (Davies et al. 2017). In another study that evaluated dog foods, Se levels in all formulas were above the legal limits determined by FEDIAF. Similar to our results, 76% (38/50) for non‐prescription, 84% (21/25) for hypoallergenic, 80% (20/25) for GI, 100% (25/25) for hepatic and 68% (17/25) for renal dog foods revealed non‐compliance with FEDIAF Guidelines (Or et al. 2024). These results reflect the need for reconsideration of the upper limits for Se in the guidelines.

### 4.5 Zinc

Zinc, one of the essential elements, has various functions in the organism, including immunity, oxidative stress, DNA metabolism, neurogenesis and growth (Maret and Sandstead 2006; Prasad 2008). Zn deficiency frequently involves multiple organ systems, including growth, sexual maturation and immune function. Zinc bioavailability may be affected by chelating agents and metal ion interactions (Gu et al. 2020). It was reported that Zn propionate had a 60%–80% higher bioavailability than Zn oxide. In the same study, it was observed that the bioavailability of both compounds was depressed by the addition of calcium to the diet (Wedekind and Lowry 1998). In commercial pet foods, mostly Zn oxide is used as a Zn source. In our study, the lowest Zn level was in the N‐P food group. Also, N‐P food Zn content was significantly lower than the GI, R and O food groups. Also, the mean concentration of Zn in the N‐P group was below the minimum recommended level of FEDIAF and AAFCO (Tables 2 and 3). Its level reveals an in‐compliance with the adequate intake and recommended allowance Zn amounts of NRC (Table 4).

In this study, 4 of 18 of the GI food group (22.2%) was lower and 1 of 18 of the GI food group (5.55%) was higher than the recommended levels by FEDIAF, which reflects the 27.7% in compliance. Only one food in the R group was below the determined limit (6.66%). Four of eight of the U food group was lower (50%) and three of eight (37.5%) was higher than the determined limits. Finally, in the U group, it was observed a total of 87.5% in compliance. In the N‐P food group, 29 of 45 (64.4%) of foods were lower than the minimum recommended level. However, in the O group, Zn levels of all foods were between the minimum and maximum limit. In a similar study that evaluated the Zn value in dry cat food, the mean Zn level was measured as 57.0 (17.2–122.2) mg/1000 kcal. Davies et al. reported a mean 176 µg/kg DM Zn concentration in dry cat foods (Davies et al. 2017).

Since Zn deficiency in small animals may lead to oxidative damage, antioxidant reduction and inadequate immune defence (Pereira et al. 2021), reconsideration of Zn concentration in both prescription and non‐prescription cat foods is recommended to prevent inadequate Zn‐related health problems in cats. On the other hand, Zn toxicosis is very scarce in cats. Only one case was reported on Zn toxicosis associated with ingestion of a Zn‐containing object in a cat with haemolytic anaemia, liver enzyme activity increases, gastrointestinal signs and pancreatitis in a cat (Yu et al. 2022).

### 4.6 Magnesium, Calcium and Phosphorus

Magnesium is one of the most abundant minerals, which plays an important role in many physiological functions in both human and animal bodies (Fiorentini et al. 2021). Hypomagnesaemia‐associated clinical findings were suggested as lethargy, vomiting, generalised tremors and seizures (Smith, Hendricks, and Centola 2022). On the other hand, hypomagnesaemia has been reported to cause cardiovascular and neurological symptoms such as vomiting, hypotension, bradycardia, paralysis and depression in cats (Jackson and Drobatz 2004). Therefore, balanced dietary Mg intake is crucial. Both the lowest of all food groups and significantly lower content of Mg compared to the GI and N‐P food groups were observed in the O group. In human medicine, many studies have revealed the relationship between hypomagnesaemia and obesity (Shamnani et al. 2018; Yakinci et al. 1997; Ul Hassan et al. 2017). In veterinary medicine, no significant difference was reported between healthy and obese dogs (Cihan et al. 2023). Although significant differences were observed in our study, mean Mg levels of all food groups were above the minimum recommended level by FEDIAF, AAFCO and NRC (Tables 2, 3, 4). No maximum legal limit is available in the guidelines. In this study, while all the prescription foods have 100% compliance with the determined minimum level of guidelines, 5 of 45 of the N‐P foods (11.1%) were below the minimum recommended level.

Calcium and phosphorus both play essential roles in many biological processes. While Ca is responsible for muscle contraction, hormone secretion, the blood‐clotting cascade and the excitation of neurons, P is involved in energy metabolism, cellular signalling, nucleic acid synthesis and the stabilisation of cell membranes (Taylor and Bushinsky 2009). In veterinary medicine, hypercalcaemia and hypocalcaemia may result in life‐threatening findings. While hypercalcaemia may cause vomiting, depression, weakness, muscular and myocardial dysfunctions, cardiac arrhythmias, seizures and coagulation abnormalities, hypocalcaemia may result in muscle tremors, seizures, restlessness, hypersensitivity and disorientation in cats (Coady, Fletcher, and Goggs 2019). In this study, the highest amount of mean Ca and P were measured in the N‐P food group. The P level in the R group was significantly lower than the N‐P group. Since seconder hyperphosphataemia is a very common finding in cats with renal failure, veterinarians are commonly prescribed a low‐phosphorus‐containing diet such as renal—kidney prescription diets. Therefore, low P content is expected in these food formulas. In our study, the mean Ca concentration of all food types was above the minimum recommended level by FEDIAF (Table 2). However, the Ca ratio in the U and O groups was slightly lower than the minimum recommended level by AAFCO (Table 3). While the mean P concentration of all food groups was above the minimum recommended level by FEDIAF (Table 2), the P ratio in all prescription food groups except for GI was below the minimum level determined by AAFCO (Table 3). In compliance with the guidelines for Ca was noted in one food in the O and one food in the U groups (10% and 12.5%, respectively). Three of the 15 foods in the R group and one food in the O group (20% and 10%, respectively) were below the minimum recommended level by FEDIAF.

In a similar study that evaluated the Mg, Ca and P levels in non‐prescription cat foods, it was suggested that pet food nutritional guidelines should be reconsidered due to the high number of foods with high P and low Ca results (Summers et al. 2020).

Consequently, due to their long‐term consumption, complete and balanced food production is important for cats. Manufacturers have formulated their food by taking into account the recommendations and guidelines of legal food authorities. However, inconsistencies between balanced and complete food ingredients and guidelines suggest that either food manufacturers or guidelines need to be re‐evaluated to avoid potential deficiencies. In the pet foods in the current study, all minerals and elements are nutritional additives. This suggests that the inconsistencies are a result of an enrichment/additive problem. Specific adjustments should be reconsidered by the food manufacturers and regulatory authorities to prevent element and mineral deficiencies and their negative health consequences with long‐term use. In addition, considering the last update was in 2021 by FEDIAF, guidelines need to be reconsidered based on recent reports and research. Based on the findings of this study, future research investigating the bioavailability of these elements and minerals in different food formulations and the long‐term health impacts of varying mineral concentrations of different foods formulated for feline nutrition is suggested.

## 5 Conclusion

Some element and mineral values showed significant differences between prescription and non‐prescription dry cat foods. Since it was observed that the high number of foods exceeded the upper limit or below the minimum recommended level by FEDIAF, AAFCO or NRC, both regulatory guidelines and the elemental composition of commercial pet food need reconsideration.

## Author Contributions

**Bengü Bilgiç**: writing–original draft, writing–review and editing, resources, conceptualisation. **Duygu Tarhan**: writing–original draft, methodology, validation, visualisation, investigation, data curation. **Fatma Ateş**: methodology, validation, visualisation, data curation, writing–review and editing. **Çağla Parkan Yaramış**: visualisation, data curation, writing–review and editing. **Lora Koenhemsi**: methodology, validation, resources, writing–review and editing. **Mehmet Erman Or**: methodology, supervision, investigation, writing–review and editing.

## Ethics Statement

The authors have nothing to report

## Conflicts of Interest

The authors declare no conflicts of interest.

### Peer Review

The peer review history for this article is available at https://www.webofscience.com/api/gateway/wos/peer-review/10.1002/vms3.70123.

## Data Availability

The data that support the findings of this study are available upon reasonable request to the corresponding author.
